# Supplementary material for: Optimizing Polymer Solar Cells Using Non-Halogenated Solvent Blends
Source: Polymers (Basel). 2019 Mar 22;11(3):544. doi: 10.3390/polym11030544 (PMC6473778; doi:10.3390/polym11030544)
Supplement: Supplementary file 1 [file polymers-11-00544-s001.pdf]

Supplementary Information

Optimizing polymer solar cells using non-halogenated solvent blends

Guler Kocak[1], Desta Gedefaw[1,2], and Mats R. Andersson [1,*]

[1] Flinders Institute for Nanoscale Science and Technology, Flinders University, Sturt Road, Bedford Park, Adelaide, SA 5042, Australia
[2] School of Biological and Chemical Sciences, The University of South Pacific, Laucala Campus, Suva, Fiji
* Correspondence: mats.andersson@flinders.edu.au; Tel.: +61-8-82013585

Table S1: The integrated currents from EQE compared to the Jsc from I-V measurement of corresponding devices.

Solvent	Integrated Jsc from EQE (mA/cm^2)	Jsc (mA/cm^2)
o-xylene	2.2	2.3
o-xylene + 2% MN	9.7	9.9
o-xylene + 3% PN	9.9	10.4
o-xylene + 3% DIO	8.2	8.9
o-xylene + 2% CN	5.9	6.4
